# Optimizing olfactory testing for the diagnosis of Parkinson’s disease: item analysis of the university of Pennsylvania smell identification test

**DOI:** 10.1038/s41531-017-0039-8

**Published:** 2018-01-15

**Authors:** James F. Morley, Abigail Cohen, Laura Silveira-Moriyama, Andrew J. Lees, David R. Williams, Regina Katzenschlager, Christopher Hawkes, Julie P. Shtraks, Daniel Weintraub, Richard L. Doty, John E. Duda

**Affiliations:** 1 Parkinson’s Disease Research Education, Clinical and Education Center, Corporal Michael J. Crescenz VA Medical Center, Philadelphia, PA USA; 2 Department of Neurology, Perelman School of Medicine, University of Pennsylvania, Philadelphia, PA USA; 3 CCEB, Perelman School of Medicine, University of Pennsylvania, Philadelphia, PA USA; 4 Department of Psychiatry, Perelman School of Medicine, University of Pennsylvania, Philadelphia, PA USA; 5 Smell and Taste Center, Perelman School of Medicine, University of Pennsylvania, Philadelphia, PA USA; 6 UCL Institute of Neurology, Monash University, Melbourne, VIC Australia; 7 Department of Medicine, Monash University, Melbourne, VIC Australia; 8 Karl Landsteiner Institute for Neuroimmunological and Neurodegenerative Disorders, Medical University of Vienna, Vienna, Austria; 9 Barts and the London School of Medicine and Dentistry, London, UK

## Abstract

The 40-item University of Pennsylvania Smell Identification Test (UPSIT) is an effective instrument to detect olfactory dusfunction in Parkinson’s disease (PD). It is not clear, however, whether tests of this length are necessary to detect such dysfunction. Several studies have suggested that detection of certain odors is selectively compromised in PD, and that a test comprised of these odors could be shorter and more specific for this purpose. Therefore, we attempted to identify a subset of UPSIT odors that distinguish PD from controls with similar or improved test characteristics compared to the full test. The discriminatory power of each odor was examined using UPSIT data from a discovery cohort of 314 PD patients and 314 matched controls and ranked using multiple methods (including odds ratios, regression coefficients and discriminant analysis). To validate optimally discriminant subsets, we calculated test characteristics using data from two independent cohorts (totaling 306 PD and 343 controls). In the discovery cohort, multiple novel 12-item subsets (and the previously described Brief Smell Identification Test-B) performed similarly or improved upon the UPSIT and were better than 12 random items. However, in validation studies from independent cohorts, multiple subsets retained test characteristics similar to the full UPSIT, but did not outperform 12 random items. Differential discriminatory power of individual items is not conserved across independent cohorts arguing against selective hyposmia in PD. However, multiple 12-item subsets performed as well as the full UPSIT. These subsets could form the basis for shorter olfactory tests in the clinical evaluation of Parkinsonism.

## Non-motor symptoms: sniffing for a faster diagnosis

A short test of olfactory function could aid the early diagnosis of Parkinson’s disease (PD). An international study led by James F. Morley at the University of Pennsylvania, USA, examined the ability of 314 PD patients and 314 matched controls to perceive 40 odors in the University of Pennsylvania Smell Identification Test (UPSIT). They used various statistical methods to determine whether tests based on a subset of these odors could distinguish PD patients from controls. Although they were not able to identify a consistent subset of smells that differentiates patients from controls, which supports the idea that there is not a PD-specific pattern of smell loss, they demonstrate that a shorter 12-item version of the UPSIT is specific and sensitive enough to detect olfactory dysfunction in PD.

## Introduction

Olfactory impairment is a common finding in Parkinson’s disease (PD), with estimates of prevalence ranging from 50% to more than 90%.^1–6^ Neurons of the olfactory system are among the first to display PD-related Lewy pathology and clinical anosmia or hyposmia may be detected years before motor symptoms present, suggesting that olfactory impairment may be one of the earliest manifestations of synucleinopathy.^7–9^ Whether or not such pathology causes olfactory dysfunction is unknown, as other explanations for the early deficits are possible.^10^ The high prevalence, persistence throughout disease, and ease of olfactory testing has fostered interest in the use of olfaction as a biomarker for early diagnostic strategies, differential diagnosis and prediction of clinical outcomes of PD and related diseases.^11^

Numerous tests have been used to measure olfactory function in PD with odor identification tests being the most common.^12–15^ Among the best-characterized and robust of such tests is the University of Pennsylvania Smell Identification Test (UPSIT).^16^ The UPSIT is comprised of four booklets, each of which contains 10 pages. An odorized “scratch & sniff” label is present on each page of each booklet. The subject scratches the label and then indicates which of four response alternatives best matches the perceived smell. The UPSIT is a robust measure of olfactory dysfunction in PD and has been described in numerous studies.^17^ However, use of the UPSIT (and other well-characterized methods such as “Sniffin Sticks”^18^) can be limited by difficulty of incorporating such a test into routine clinical encounter. Shorter tests would seem to be preferable both from the perspective of the patient and the neurologist, particularly within a busy clinical setting.

Shorter tests have indeed been developed, although, as noted in the discussion, there is a trade-off between test length, sensitivity, and reliability. Among such tests is the 12-item Brief Smell Identification Test (B-SIT),^19,20^ whose test items, derived from the UPSIT, were designed to be cross-cultural in familiarity. This test has been used to assess the prevalence of, or conversion to, such neurodegenerative diseases as Alzheimer’s disease (AD)^21,22^ and PD^23^ and has several parallel forms. These forms include odors and response alternatives potentially more sensitive to specific neurodegenerative diseases [e.g., B-SIT Version A for AD based upon^24^ and B-SIT Version B for PD based upon.^25^ Numerous other brief screening tests also have been developed, including ones using as few as two odors, although not all have been administered to neurodegenerative disease populations. These include members of the 3-item and 4-item Pocket Smell Test^TM^ series (PSTs),^26,27^ the 3-item Quick Smell Identification Test^TM^ (Q-SIT),^28^ a 3-item version of the Sniffin’ Sticks test,^29,30^ and a 2-item version of the Open Essence Smell Identification Test,^31^ a recent modification of the more widely used Japanese Odor Stick Identification Test.^32^ Similarly, subsets of other well-characterized olfactory tests (Sniffin Sticks) have been proposed as shorter and more convenient assays for clinical screening.^29,30^

In addition to the development of briefer tests is the question as to whether a pattern of smell loss can be identified that is more specific to PD relative to aging or other disorders that impact smell function. Double and colleagues identified a set of 5 B-SIT items that correctly differentiated 82% of PD cases (ref. ^33^, and an early study by Hawkes suggested that 2 UPSIT items alone could effectively distinguish PD patients from controls.^34^ Bohnen and colleagues identified three odors that were 75% accurate in differentiating PD from controls and were better correlated with dopamine transporter imaging than total UPSIT score.^35^ Other studies using Sniffin Sticks have similarly proposed odors that are selectively affected in PD compared to other causes of hyposmia including head trauma or aging.^29,36^ However, as summarized in Table 1, the putative “PD-specific” items vary widely across studies, raising questions about their reliability and validity in the wider PD population. Other studies have found no such selectivity (refs. ^6,37^ Whether there is a selective pattern of hyposmia in PD that can be observed across different cohorts is an unanswered question that has important implications for the development of shorter, more sensitive and specific assays.

**Table 1:** Examples of currently available olfactory tests used in PD and previously proposed discriminant subsets of odors

Test/Author	# Odors	Comment	Ref
Scratch and Sniff-based			
UPSIT	40	Odor identification. Used in >100 PD studies	50
B-SIT	12	Designed to be shorter and cross-culturally valid. Not intended to be PD specific	19
B-SIT-B	12	Based on the BSIT. Modified with the intention to be more specific for PD	25
Double	5	Gasoline, banana, pineapple, smoke, cinnamon Identified 82% of PD cases correctly	33
Pocket Smell Test	3	Lemon, lilac, smoke. Not intended to be PD -specific	26
Bohnen	3	Banana, licorice, dill pickle. 75% accurate in identifying PD. Better correlated with dopamine transporter imaging than total UPSIT score	35
Hawkes	2	Pizza, wintergreen. 90% sensitivity 86% specificity for PD	34
Odor pen-based			
Sniffin’ Sticks	16	Odor identification (modules for threshold and discrimination as well). Well-characterized in PD	18
Mahlknecht	8	Licorice, anise, mint, cinnamon, banana, pineapple, rose, coffee. 84% sensitivity, 88% specificity for PD	29
Casjens	3	Coffee, peppermint, anise. Similar misclassification for rate for PD compared to using 16 odors	36
Hummel	3	Cloves, coffee, rose. 96% sensitivity and 66% specificity for olfactory dysfunction in the general population. Not intended to be PD-specific	30

*UPSIT* University of Pennsylvania smell identification test, *B-SIT* brief smell identification test, *B-SIT-B* brief smell identification test, version B

The objective of this study was to determine whether a shorter version of the UPSIT could be developed that retained or improved the sensitivity and specificity in detecting hyposmia in PD. Our approach was to comprehensively analyze the discriminatory power of individual UPSIT items using a variety of statistical methods to identify subsets of odors that robustly distinguish PD patients from controls. We first derived candidate subsets in a large matched discovery cohort and then examined their performance in two independent populations of PD patients and controls.

## Results

### Many subsets of UPSIT items distinguish PD from controls

We first examined the test characteristics of previously proposed or commercially available subsets of UPSIT odors to distinguish PD patients from control subjects. Group means for the full 40 item-UPSIT, the 12 items comprising the B-SIT, B-SIT-B, 5 items identified by Double et al.,^33^ odors from the 3-item Pocket Smell Test, 3 items previously identified by Bohnen et al.^35^ and two items suggested by Hawkes^34^ were significantly lower in PD patients compared to controls (Table 2). Sensitivity and specificity were similar between the UPSIT and each of the 12 item tests (Table 2). The 5 item scale based on odors from Double et al., 3-item subsets and the 2 items proposed by Hawkes had lower sensitivity and/or specificity compared to the full test (Table 2).

**Table 2:** Different sets of odors distinguish between PD and control subjects

	Items	Control mean (SD)	PD mean (SD)	*p*	AUC (95% CI)	Sen	Spe	Cut
UPSIT	40	28 (8.7)	19 (7.2)	<0.001	0.78 (0.74–0.82)	0.84	0.66	27
BSIT	12	8.7 (2.7)	5.8 (2.6)	<0.001	0.78 (0.74–0.82)	0.85	0.62	9
BSIT-B	12	8.3 (3.0)	5.0 (2.3)	<0.001	0.80 (0.76–0.83)	0.86	0.67	8
Double	5	3.5 (1.3)	2.2 (1.4)	<0.001	0.75 (0.71–0.79)	0.79	0.58	4
Bohnen	3	2.0 (1.0)	1.0 (0.85)	<0.001	0.75 (0.71–0.79)	0.73	0.70	2
PST	3	2.2 (0.89)	1.6 (1.0)	<0.001	0.69 (0.65–0.74)	0.78	0.54	3
Hawkes	2	1.3 (0.75)	0.85 (0.75)	<0.001	0.67 (0.63–0.71)	0.79	0.50	2

Data are mean(SD) of the number of correctly identified odors, Area under the receiver-operator characteristic curve (AUC), sensitivity (Sen) and specificity (Spe). Cut = cut-off number of correct answers used for point sensitivity and specificity

*UPSIT* University of Pennsylvania smell identification test, *B-SIT* brief smell identification test, *B-SIT-B* brief smell identification test, version B, *PST* pocket smell test

### Development of novel UPSIT subsets for the detection of hyposmia in PD

We attempted to identify novel subsets of UPSIT odors that might outperform the full test using different statistical ranking strategies (see methods for full details). This approach narrowed the 40 UPSIT items to a total of 22 unique items that were in the top 12 of at least 1 of the 4 initial ranking approaches (Table 3). Eleven of the items appeared on at least 3 of the 12-item lists. Four items (smoke, soap, licorice, bubblegum) appeared on all lists. Multiple 12-item subsets had test characteristics similar to the full UPSIT (Table 4). Some, such as the 12-item Combined list, had slightly better test characteristics compared to full UPSIT (Sens/Spec, 0.84/0.77 vs. UPSIT 0.84/0.71, Table 4). Relatively poorer test characteristics were observed for 12-item subsets derived at random (0.78/0.65) or from the worst ranking items (0.72/0.53, Table 4) in the discovery cohort. Further shortening the top-12 items lead to steady declines in AUC and/or the optimal combination of sensitivity and specificity as items beyond the top 11 were removed (Table 4).

**Table 3:** Putative subsets highly discriminant of UPSIT items

Rank	Difference	Odds ratio	Discriminant	Regression	Combined	Worst
1	**Smoke**	**Smoke**	Lime	Grass	**Smoke**	Rootbeer
2	Motor oil	Grass	Turpentine	Lime	Grass	Watermelon
3	Soap	Licorice	Smoke	Banana	Turpentine	Leather
4	Gasoline	Lemon	Banana	Turpentine	**Soap**	Onion
5	Paint thinner	Motor oil	**Bubblegum**	**Smoke**	Lime	Gingerbread
6	Peanut	Turpentine	Grape	**Bubblegum**	**Bubblegum**	Peach
7	Grass	Dill pickle	Soap	Cherry	Motoroil	Cheddar cheese
8	Lemon	**Bububblegum**	Dill pickle	Grape	Banana	Cinnamon
9	Wintergreen	**Soap**	**Licorice**	**Soap**	**Licorice**	Chocolate
10	Grape	Gasoline	Pine	Mint	Grape	Mint
11	**Licorice**	Lime	Cedar	Cinnamon	Lemon	Cherry
12	**Bubblegum**	Paint thinner	Gasoline	**Licorice**	Gasoline	Strawberry

Summary of subsets of UPSIT items that were identified as highly discriminant for differentiating PD from control subjects in the discovery cohort. Listed are the top 12 most discriminant UPSIT items ranked by five different methods: 1) the absolute difference in percentage of PD and control subjects answering incorrectly (Difference), 2) odds ratio, 3) discriminant function analysis (Discriminant),4) logistic regression (Regression), 5) a weighted average combining the first four methods (Combined). The worst 12 items using the difference method (Worst)are shown for comparison. To highlight odors that were found as highly discriminant for differentiating PD from controls using multiple different ranking methods, items appearing on the all of the “Difference”, “Odds Ratio”, “Discriminant”, “Regression” and “Combined” lists are shown as bold. 12 item lists were used to facilitate comparison with existing, commercially available smell tests such as the B-SIT

**Table 4:** Test characteristics of putative subsets of highly discriminant UPSIT items from the discovery cohort

	Items	AUC (95% CI)	Sensitivity	Specificity	Cut-off
UPSIT	40	0.78(0.74–0.82)	0.84	0.66	27
Difference	12	0.82(0.78–0.85)	0.77	0.74	8
Odds Ratio	12	0.81(0.79–0.86)	0.84	0.67	8
Regression	12	0.80(0.76–0.83)	0.82	0.68	8
Discriminant	12	0.81(0.78–0.75)	0.83	0.69	8
Combined	12	0.83(0.80–0.86)	0.84	0.71	8
Random	12	0.76(0.72–0.79)	0.78	0.65	8
Worst	12	0.65(0.61–0.70)	0.72	0.53	9
Items from the “Combined” List					
	11	0.83(0.80–0.86)	0.80	0.72	8
	10	0.82(0.79–0.85)	0.76	0.76	8
	9	0.81(0.79–0.85)	0.70	0.77	6
	8	0.80(0.79–0.85)	0.75	0.74	6
	7	0.80(0.78–0.85)	0.85	0.67	5
	6	0.79(0.76–0.84)	0.80	0.70	4
	5	0.79(0.76–0.84)	0.67	0.74	3
	4	0.78(0.75–0.82)	0.71	0.70	3
	3	0.77(0.74–0.81)	0.68	0.74	2
	2	0.73(0.70–0.77)	0.84	0.57	2

Data are area under the receiver-operator characteristic curve (AUC), sensitivity and specificity for differentiating PD from control subjects in the discovery cohort. Cut-off = number of correct answers used to determine the point sensitivity and specificity. The subsets of highly discriminant items were determined by ranking odors using five different methods: 1) the absolute difference in percentage of PD and control subjects answering incorrectly (difference), 2) odds ratio, 3) discriminant analysis (discriminant), 4) logistic regression (regression), 5) a weighted average combining the first four methods (combined). For comparison, test characteristics for 12 random items and the worst 12 items using the difference method (worst) are shown. 12 item lists were used to facilitate comparison with existing, commercially available smell tests such as the B-SIT. In the second half of the Table, test characteristics for subsets containing decreasing numbers of the 12 most highly discriminatory items from the discovery cohort are shown

*UPSIT* University of Pennsylvania smell identification test

### “PD-specific” subscales derived in one population do not retain discriminatory power across independent cohorts

We examined the performance of our putative PD-specific subsets with individual item UPSIT data from two independently derived validation cohorts. As in the discovery cohort, test characteristics including sensitivity, specificity and AUC for multiple 12-item subsets were similar to those for the UPSIT indicating that smaller subscales can maintain comparable discriminatory power (Figure). However, when tested in the independent samples, the most highly discriminatory subsets from the discovery cohort did not perform better than a random subset or, in fact, the worst ranking 12 items derived from the discovery cohort. For example, AUCs for the Combined-12 subset, full UPSIT and Worst-12 subset calculated with data from the discovery cohort were 0.83 (95% CI 0.80–0.87), 0.78 (95% CI 0.74–0.82), and 0.66 (95% CI 0.62–0.74) respectively (Tables 2, 3). Using data from the Barts cohort, however, these values were essentially identical to one another (AUCs: Combined-12 = 0.85, UPSIT = 0.87, Worst-12 = 0.86, Figure). Similar results were observed using data from the UCL cohort (AUCs: Combined-12 = 0.82, UPSIT = 0.90, Worst-12 = 0.87, Figure).

### Effect of age and gender on olfactory test performance

As age and sex are important determinants of olfactory function, we examined the test characteristics of the UPSIT, B-SIT-B and 12 UPSIT items (“Combined” list) we defined as most highly discriminatory in the discovery cohort (Table 5) as a function of age and sex using data from all three cohorts (*N* = 1279). We found that although the test AUCs were fairly similar in men and women, higher cut-off values were required for optimal sensitivity/specificity in women. Additionally, the tests effectively distinguished between PD and controls in all age groups but, generally, we observed higher AUCs in subjects less than 74 years old (Table 5). The pattern of age/sex influence was similar across the different tests.

**Table 5:** Effect of age and sex on olfactory test characteristics

Test	Age	AUC (95% CI)		Cut-off		Sensitivity		Specificity	
		Men	Women	Men	Women	Men	Women	Men	Women
UPSIT	<63	0.86 (0.82–0.90)	0.81 (0.75–0.87)	27	29	0.79	0.74	0.83	0.79
	63–73	0.88 (0.84–0.92)	0.88 (0.83–0.94)	23	26	0.83	0.83	0.79	0.86
	>73	0.76 (0.70–0.82)	0.82 (0.71–0.93)	23	24	0.78	0.80	0.68	0.83
BSIT-B	<63	0.85 (0.81–0.90)	0.80 (0.74–0.87)	8	9	0.80	0.71	0.80	0.77
	63–73	0.87 (0.83–0.91)	0.88 (0.82–0.94)	7	8	0.89	0.85	0.75	0.85
	>73	0.77 (0.71–0.83)	0.82 (0.71–0.93)	7	8	0.78	0.87	0.68	0.72
Combined	<63	0.85 (0.81–0.89)	0.76 (0.70–0.83)	8	9	0.74	0.67	0.81	0.74
	63–73	0.86 (0.82–0.91)	0.86 (0.80–0.92)	7	8	0.79	0.82	0.77	0.77
	>73	0.78 (0.72–0.84)	0.82 (0.72–0.92)	7	7	0.78	0.77	0.63	0.72

Data are area under the receiver-operator characteristic curve (AUC), sensitivity and specificity for differentiating PD from control subjects in the discovery cohort. Cut-off = number of correct answers used to determine the point sensitivity and specificity. Subjects from all three cohorts (*N* = 1279) were divided by sex and age tertile (<63 years old, 63–73 years old and >73 years old).* UPSIT* University of Pennsylvania smell identification test, *BSIT-B* brief smell identification test, version B. Combined: Top 12 items found most highly discriminatory in the discovery cohort

## Discussion

Detecting anosmia or hyposmia is of significant interest for early identification and differential diagnosis of PD and related disorders. Although the 40-item UPSIT has been found to be an effective instrument to detect anosmia or hyposmia in PD, it is not clear whether tests employing fewer UPSIT items are equally useful in detecting such olfactory dysfunction. Several studies have suggested that certain odors are selectively compromised in PD, and that a test comprised of these odors could be shorter, easier to administer, and more specific for this purpose. However, little uniformity exists across studies. Some of the candidate subsets identified using “scratch and sniff” tests (UPSIT, B-SIT versions) include gasoline, banana, pineapple, smoke and cinnamon,^33^ licorice, banana, dill pickle^35^ and wintergreen and pizza^34^ (Table 1). Studies using Sniffin Sticks have similarly proposed subsets of highly discriminant odors including coffee, peppermint and anise,^36^ cloves, coffee and rose,^30^ and a recent study identified set of 8 (of 16) Sniffin Sticks that had excellent diagnostic accuracy for early PD and even correctly identified subjects with idiopathic REM-sleep behavior disorder who went on to develop PD.^29^ While some odors have been suggested by multiple groups (smoke, coffee, banana, licorice), none have been reported uniformly.

When we used four different methods to assess discriminatory capacity, 4 items appeared among the top 12 on all of the lists (Table 3). By chance alone, one would expect less than one item to appear on all lists, suggesting the possibility that these methods did enrich for more highly discriminatory odors in the discovery cohort. Poorer test characteristics were observed for 12-item subsets derived at random or from the worst ranking items (Table 4) compared to the highest ranking items in accord with the idea that we may have identified subsets with greater discriminatory power. However, when examined in two independent validation cohorts, the putative highly discriminant subsets performed no better than randomly selected items or even the least discriminatory items from the discovery cohort (Figure).

Support for our overall findings of a lack of a consistent small subset of odorants that differentiates PD patients from controls comes from an item analysis performed on the UPSIT in 1988.^6^ In this study, the pattern of responses of 81 PD patients (based upon the proportion of persons correctly answering an item) across the 40 items of the UPSIT was similar to that of 81 matched controls (Spearman rank order correlation across odor items = 0.75), suggesting the deficit is a general one and unlikely confined to any subset of UPSIT items.

There are several reasons why a uniform set of odorants specific to PD has not been found. First, it is conceivable that such a set is not detected because it is overshadowed by the variability derived from cultural or other differences between populations that have been previously studied.^20,38–40^ Second, the lack of specificity to PD may reflect the absence of specific damage to different receptor classes or receptor channels, either at the level of the epithelium or at higher levels within the central nervous system, including the olfactory bulb. The human olfactory nerve is comprised of 6–10 million olfactory receptor cells, of which there are nearly 400 types harboring G-protein coupled odor receptors (GPCRs) on their cilia, with a given cell expressing only one type of receptor. In most cases, each receptor responds to a range of odorants, such that even a single chemical can stimulate multiple sets of receptor cells. Even if some subset of receptors were damaged specifically by PD, the gestalt of a given smell, like the perception of visual objects, can likely resist the loss of some segments of the olfactory “object” and still retain identification ability via feature-detection processes.^31^ Third, the search for odorants specific to PD is further complicated by the fact that most if not all of the odorants employed in the extant olfactory tests are comprised of multiple chemicals. Until there is a better understanding of the relative distribution numbers of the ~400 classes of receptor types within the epithelium and the nature and range of ligands that activate each receptor type, finding sets of odorants that might be specifically damaged by PD or any other disease is unlikely. Finally, the quest is further confounded by attempting to compare results across studies using different tests with seemingly the same “odors”. Even if the qualitative “odor” from one test appears to be the same qualitative “odor” as that from another, different chemicals and combinations of chemicals can make up the same “odor”. In other words, different odorants or combinations of odorants often are being compared.

While the large number of subjects in the discovery cohort and use of multiple independent validation cohorts are strengths, this study has several limitations that are important to consider. Most patients involved in the study were not autopsy verified so that some of the PD subjects likely had non-Lewy body Parkinsonism and some of the controls may have had pre-motor PD other conditions associated with olfactory dysfunction.^41–43^ Similarly, in many cases, these were subjects with well-established PD and it is not clear that our results can be generalized to patients with early or de novo PD. Our analysis of individual items and novel combinations was retrospective using existing UPSIT data and, therefore, cannot account for item ordering or the effect of distractor choices that would be present if the proposed UPSIT subsets had been presented together as independent tests. Smoking history was not available for all subjects, but smoking has a relatively small impact on olfactory function, compared to factors such as age, sex or the presence underlying neurologic disease, such as PD (ref. ^44^). Indeed, age and sex are significant determinants of olfactory function such that optimal UPSIT cut-off scores can differ between men and women or among different age groups.^45^ Similarly, we found that higher cut-off values were required for optimal sensitivity/specificity in women, reflecting generally better olfactory performance compared to men. The tests effectively distinguished between PD and controls in all age groups but performed best in subjects less than 74 years old. However, the influence of age and sex were similar using the full-length UPSIT or subsets of UPSIT items (Table 5).

**Fig. 1:**
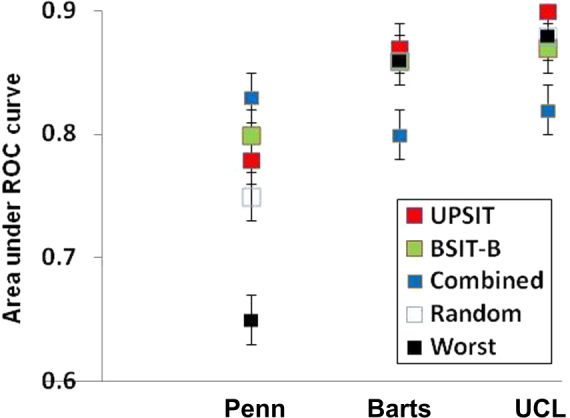
Novel UPSIT subsets do no retain discriminatory power across independent cohorts. Data are area under the receiver-operator characteristic curve (error bars represent the 95% confidence interval) for the 40 UPSIT items (red), or items from the BSIT-B (green), “combined” subset (blue), random 12 items (white) or worst 12 items (from the training cohort) when tested using data from the training cohort (Penn) or two independent cohorts (Barts, UCL)

Finally, this study examined multiple international cohorts for discovery and validation but only included subjects from the US and UK. Cultural factors influencing recognition of certain odors are known to affect performance on olfactory identification tests in other populations, possibly limiting generalizability of these results to other cultures.^20,38–40^ Similarly, cultural heterogeneity between the discovery and validation cohorts could explain some of the variable performance of different subsets of UPSIT items between the cohorts (Fig. 1).

While our results, along with those of earlier studies, argue against selective anosmia or hyposmia in PD, they do suggest that shorter versions of the UPSIT or Sniffin’ Sticks retain much of the discriminatory power of the full tests for detecting olfactory dysfunction in PD. The decision to employ a short or long test for a given clinical or research purpose depends on a number of factors, including the setting of the administration, proposed indication, and pre-test probability of PD in the population studied. As discussed in detail, shorter tests may maintain suitable test characteristics for a binary outcome (diagnosis). However, longer tests are more sensitive to subtle alterations in function and allow for distinctions between degrees of dysfunction, which can be critical for counseling patients regarding prognosis, including patients with non-neurodegenerative disorders such as head trauma.^46^ Longer tests also allow for the detection of malingering on the basis of improbable forced-choice responding,^47^ which cannot be discerned from shorter tests, and are clearly more reliable than shorter tests.^48^ We found that decreasing even the most discriminatory set of items to fewer than 11 odors resulted in steadily decreasing test performance. This can have an impact when small samples are being tested or when individual patients or subjects are being assessed. It must be kept in mind, of course, that while olfactory testing can be a very sensitive aid in diagnosing PD, e.g., in differentiating between PD from progressive supranuclear palsy and essential tremor, it is not specific to PD.^10^

Our findings that 12-item UPSIT subsets performed better that the full 40-item test in a discovery cohort but not in independent replication cohorts has several practical implications for the use of olfactory tests for PD. First, 12-item tests are sufficient and may save time and cost compared to the full UPSIT. Second, attempts to discover new “PD-specific” odor sets may be ill-advised as they can be defined by chance in any cohort but are unlikely to generalize to the broader PD population. Further, we found that AUC, sensitivity and specificity declined as items were removed from the 12-item subsets suggesting that significantly shorter tests would lack sufficient diagnostic utility. Additionally, any such shorter or “PD-specific” test would lack normative data for categorizing individual patients and would need prospective validation in new cohorts. Overall, the balance of evidence suggests that shorter versions of the UPSIT—particularly the currently available B-SIT-B—should be employed with confidence to allow decreased time of administration and cost of olfactory assessment in a variety of clinical and research applications for the evaluation of Parkinsonism.

## Methods

### Subjects and olfactory assessment

For the initial (discovery cohort) phase of the UPSIT item analysis, we examined individual UPSIT item results from a convenience sample of PD patients (*N* = 314) and age-matched controls (*N* = 314) that had been administered in several protocols at the Michael J. Crescenz VA Medical Center in Philadelphia and the University of Pennsylvania. The mean (SD) age in each group was 67.4 (10.0) years and each was comprised of 83% males and were 94% Caucasian. Among PD patients, the median(interquartile range) Hoehn and Yahr stage and mean (SD) UPDRS motor scores were 2(2–3) and 22 (10.1), respectively. In an attempt to validate the performance of putative PD-specific UPSIT subsets, we used individual item data from two independent validation cohorts of PD patients and control subjects derived at University College, London (UCL Cohort) and Barts & The London School of Medicine and Dentistry (Barts Cohort). The Barts cohort was comprised of 176 PD patients with a mean age 60 (9.8) years and 177 control subjects with a mean age of 62 (10.7) years (*p* = 0.15). Subjects in the Penn cohort were only 6% non-white. Race data were not collected for all of the Barts/UCL subjects but they were largely drawn from the Oldchurch /Queens and UCL hospital patients. The vast majority were middle class Caucasian British. There were 167 PD subjects (mean age = 63 (9.9)) and 130 controls (mean age = 65(9.5)) in the UCL cohort. Most subjects were screened extensively for nasal disease. However, some subjects, particularly controls that were tested in community settings such as malls or state fairs, did not undergo rigorous screening, though subjects with clear active rhinitis of any etiology were not included. All studies from which UPSIT results were analyzed were approved by Institutional Review Boards (IRB) at Cresencz VA Medical Center, University of Pennsylvania, Barts and The London School of Medicine and Dentistry and University College London. Methods were performed in accordance with relevant regulations and guidelines. Informed consent was obtained from patients before participation in protocols.

### Statistical analysis

Individual responses to each of the 40 items were recorded as correct or incorrect. Discriminatory power of individual odors to differentiate between PD patients and control subjects was tested using several statistical approaches. First, individual odors were ranked by the difference between the percentages of PD patients versus controls answering incorrectly (Difference). A complimentary approach ordered odors by odds ratio of PD versus controls grouping for each item (Odds Ratio). The third method used discriminant function analysis, a method based on ANOVA that generates models incorporating all items into one or more weighted functions to come up with two sets, one that best discriminated PD versus controls (Discriminant) and one that least discriminated PD versus controls (Worst).^49^ We also used logistic regression to identify items that best explained variation in outcome using diagnosis of PD versus controls as the dependent variable and ranking individual odors by the associated beta-coefficient (Regression). Finally, we generated a fifth list (Combined) using a weighted matrix by taking the top-12 items identified by each of the four methods (see Table 2), assigning 12 points for highest rank, 11 for second, 10 for third, etc. and summing the score for each item, in an attempt to capture items identified in common with the different statistical approaches. A random list of twelve items (Random) was assembled by using a random number generator taking integers 1–40 and using the first 12 corresponding UPSIT items based on their order of presentation during the full test. Twelve odor subsets were chosen to facilitate comparison with several commercially available tests also containing 12 items (Table 1). Test characteristics including sensitivity (number of PD subjects scoring below the cut-off value/total number of PD subjects), specificity (number of PD subjects scoring below the cut-off value/(number of PD + control subjects scoring below the cut-off value) and area under the receiver-operator characteristic curve (AUC) were calculated for candidate subsets. Cut-offs for point sensitivities and specificities were chosen to maximize the sum of both values.

Statistical analyses were conducted using SPSS version 17.0 (SPSS Inc.; Chicago, IL). All statistical tests were two-sided and significance was set at the 0.05 level. Data that support the findings of this study are available from the corresponding author upon request.

### Disclaimer

The views expressed in this article are those of the authors and do not necessarily reflect the position or policy of the Department of Veterans Affairs or the United States government
